# Atteinte pulmonaire sévère au cours de la vascularite hypocomplémentémique urticarienne

**DOI:** 10.11604/pamj.2016.24.285.8168

**Published:** 2016-07-28

**Authors:** Mohammed Raoufi, Mustapha Laine, Hicham Naji Amrani, Hicham Souhi, Hicham Janah, Hanane Elouazzani, Ismail Abderrahmane Rhorfi, Ahmed Abid

**Affiliations:** 1 Service de Pneumologie, Hôpital Militaire Mohamed V, Rabat, Maroc

**Keywords:** Mc duffie, hypocomplémentémie, dyspnée, Mac Duffie, hypocomplementemia, dyspnea

## Abstract

L'atteinte pulmonaire au cours de la vascularite urticarienne hypocomplémentémique (VUH) ou syndrome de Mc duffie est très rare et de pronostique péjoratif. Nous rapportons le cas d'une patiente âgée de 55 ans suivie pour VUH depuis 20 ans. Le diagnostic était retenu sur les lésions urticariennes, l'inflammation occulaire, le test positif C1q-p par immunodiffusion, avec un faible du taux de C1q. La patiente a été traitée par des cycles à base de cyclophosphamide, des corticoïdes et des cures de rituximab alors qu'elle a développé une dyspnée actuellement classe III (classification NYHA). Le bilan clinico-radiologique et fonctionnel a montré une distension thoracique et une broncho-pneumopathie obstructive sévère non amélioré par le traitement systémique. Un traitement par aérosolthérapie a été démarré et la patiente présentait une nette amélioration clinique. L'atteinte pulmonaire au cours de la vascularite urticarienne hypocomplémentémique de Mc duffie conditionne le pronostique vital à cours terme. La connaissance des différentes formes de cette atteinte ouvre de nouvelles perspectives thérapeutiques.

## Abstract (en)

Pulmonary involvement in hypocomplementemic urticarial vasculitis (HUV) or Mac Duffie syndrome is extremely rare with a poor prognosis. We report the case of a 55-year-old female patient treated for HUV over a period of 20 years. The diagnosis was confirmed on the basis of urticarial lesions, ocular inflammation, positive C1q-p test by immunodiffusion, with low rate of C1q. The patient was treated with cycles of cyclophosphamide, corticoids and rituximab as she developed class III dyspnea (NYHA classification ). The clinico-radiological and functional assessment showed thoracic distension and severe obstructive pulmonary disease which found no significant improvement with systemic treatment Aerosol therapy was started and the patient had a marked clinical improvement. Pulmonary involvement in Mac Duffie hypocomplementemic urticarial vasculitis worsens the patient short-term vital prognosis. The knowledge of the different types of pulmonary involvement opens new therapeutic prospects.

## Introduction

La vascularite urticarienne hypocomplémentémique (VUH) ou syndrome McDuffie, est une maladie relativement rare qui a été rapportée principalement durant la quatrième décennie de la vie. Elle est caractérisée par une vascularite urticarienne récurrente, arthralgie et un faible taux sérique du complément. Un angio-œdème, une inflammation oculaire, une glomérulonéphrite et une pneumopathie obstructive peuvent être observés au cours de cette maladie.

## Patient et observation

**Figure 1:**
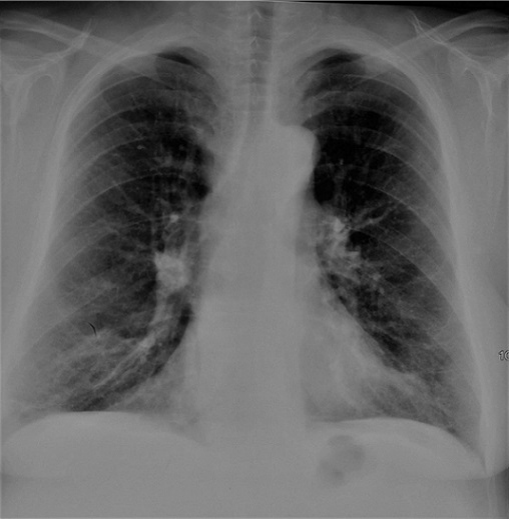
Radiographie thoracique de face montrant des signes de distension thoracique et un épaississement bronchique

**Figure 2:**
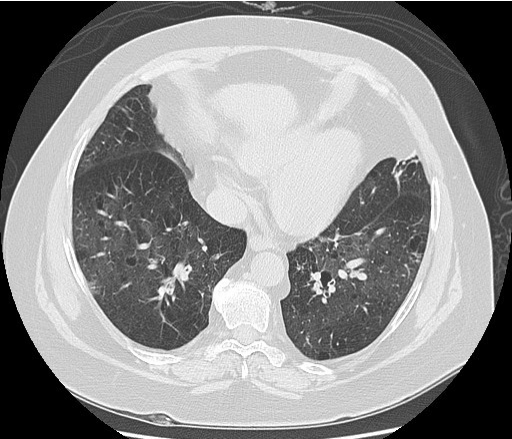
TDM thoracique montrant un emphysème centrolobulaire avec plages de verre dépoli diffuse

**Figure 3:**
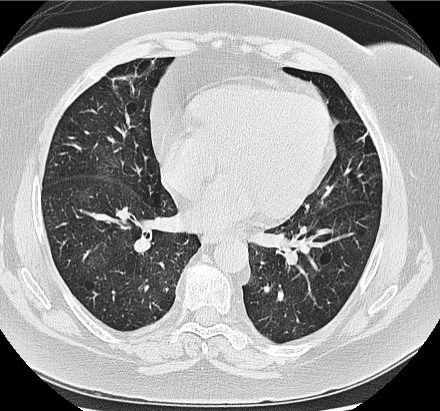
TDM du thorax montrant une majoration du diamètre cranio-caudal du thorax, et une rarification parenchymateuse

Il s'agit d´une patiente âgée de 55 ans suivie pour vascularite urticarienne hypocomplémentémique depuis 20 ans en médecine interne, retenue sur un faisceau d'arguments (lésions urticariennes récurrentes, hypocomplémentémie sérique, uvéite, dyspnée). Un traitement par cycles à base de cyclophosphamide, des corticoïdes et des cures de rituximab a été démarré, avec une régression des lésions urticariennes, mais sans amélioration de la dyspnée. En consultation de pneumologie, l'examen clinique a montré la présence de râles sibilants bilatéraux diffus. La radiographie thoracique de face a montré des signes en faveur d'une distension thoracique et un syndrome bronchique bilatéral (Figure 1). Le bilan biologique (ionogramme sanguin, la protidémie, la glycémie, l'osmolarité sanguine, le bilan lipidique, la numération sanguine, l'électrophorèse des protides) était normal. La tomodensitométrie du thorax a montré une distension thoracique, et de multiples bulles d'emphysème para-septal et centrolobulaires bilatérales, ainsi que des plages de verre dépoli (Figure 2, Figure 3). La Spiromètrie a montré un trouble ventilatoire mixte avec prédominance obstructive (VEMS à 22% de la valeur théorique, CVF à 35% de la valeur théorique, VEMS/CVF à 53). Étant donné que la thérapie du syndrome n'a pas montré une amélioration de la fonction respiratoire, un traitement par salbutamol et fluticasone a été donné en vue d'un freinage du déclin du VEMS chez la patiente. Un control un an après, n'a pas montré une grande différence en matière de VEMS ou de la CFV.

## Discussion

La vascularite urticarienne hypocomplémentémique est une maladie rare (une centaine de cas rapportés dans la littérature), qui atteint préférentiellement la femme jeune, caractérisée par une urticaire récidivante et ayant les caractères histologiques d'une vascularite leucocytoclasique. Elle est associée à une hypocomplémentémie due à la présence d'anticorps dirigé contre la fraction C1q du complément. Les critères diagnostiques de cette affection ont été proposés par Schwartz et al. Et sont indiqués dans le Tableau 1 [1]. L'abaissement du taux sérique du C1q est également un élément diagnostique important. Le diagnostic positif repose sur l'association de deux critères mineurs au moins et aux deux critères majeurs, les critères d'exclusions sont : cryoglobulinémie significative, la présence d'AC anti DNA natif, un titre élevé d'AC anti nucléaire. Un déficit héréditaire du complément et le déficit en inhibiteur de la protéine C1. L'atteinte pulmonaire au cours de la vascularite urticarienne hypocomplémentémique se manifeste par la dyspnée, la toux, et même l'hémoptysie. L'épanchement pleural peut survenir, mais la broncho-pneumopathie obstructive reste de loin la plus fréquente, elle est présente dans 50 % des cas [2]. La prévalence de précipitines C1q dans la vascularite urticarienne hypocomplémentémique est de 80 à 100 % et peut être associée à une plus grande fréquence à la BPCO. L'emphysème est retrouvé dans plus de la moitié des cas chez les patients atteints, et les symptômes peuvent être absents. Selon Jones et al, l'emphysème a un début précoce (Souvent avant l'âge de 30 ans), et peut être cliniquement significatif dans les deux ans suivant le diagnostic [3, 4]. La BPCO est progressive dans la vascularite urticarienne hypocomplémentémique, elle est considérée comme la cause la plus fréquente du décès [5]. Le tabagisme accélère nettement le développement de l'emphysème au cours de la vascularite urticarienne hypocomplémentémique, mais celui-ci peut se produire même en dehors du tabagisme. La maladie pulmonaire de type restrictif survient dans moins de 25 à 30 % chez les patients atteints de lupus systémique. Les patients sont également prédisposés aux infections à pyogènes, y compris les pneumonies [6]. Les autres atteintes systémiques sont résumées dans le Tableau 2 [7]. Selon les données de la littérature, les patients atteints de la VUH avec un taux sérique du complément peu effondré nécessitent seulement un traitement symptomatique. Le médicament de choix pour le traitement des lésions cutanées est un antihistaminique. Les antihistaminiques servent uniquement à contrôler le prurit, et sont généralement insuffisantes, car ils interviennent à la fin de la cascade de l'inflammation [8]. La formation des complexes immuns n´est pas contrôlée, et l'évolution de la maladie n´est pas arrêtée.

**Tableau 1:** Critères diagnostiques de la vascularite hypocomplémentémique urticarienne

Critères majeurs	Critères mineurs
Urticaire chronique évoluant depuis plus de six mois	Vascularite atteignant les veinules du derme à l’examen histologique
Hypocomplémentémie	Arthralgies ou arthrite
	Douleurs abdominales récidivantes
	Uvéites ou épisclérite
	Glomérulonéphrite
	Anticorps anti C1q

**Tableau 2:** Atteintes systémique de la vascularite hypocomplémentémique urticarienne

Organe ou système	Pourcentage d’atteinte (%)	Type d’atteinte
peau	100	Exanthème urticarien, vascularite urticarienne, purpura, angio-œdème
articulations	70	Arthralgies, arthrites
reins	50	Protéinurie, hématurie, insuffisance rénale, glomérulonéphrite rapidement progressive
tractus gastro- intestinal	30	Douleurs abdominale, nausées, vomissement, diarrhées, hépatomégalie, splénomégalie
yeux	10	Episclérite, uvéite, conjonctivite
cœur	RARE	Péricardite, valvulopathies
système nerveux	RARE	Tumeurs cérébrales, méningites aseptiques, neuropathies périphériques

Il n´existe aucun traitement spécifique pour la vascularite urticarienne hypocomplémentémique. Si plusieurs traitements ont été essayés, aucun consensus quant à un régime thérapeutique efficace n'a été mis en place. Des anti-inflammatoires non stéroïdiens pour le traitement symptomatique de la douleur articulaire peuvent être utiles. L'atteinte pulmonaire, ou d´autres organes peut se produire, ce qui peut nécessiter des traitements spécifiques pour la maladie notamment pour les phases aigues d'immunodépression. Ainsi, les décisions de traitement doivent être individualisées selon le statu clinique du patient. Certains cas répondent aux traitements couramment utilisés dans le traitement du lupus systémique, comme la prednisone à faible dose, l'hydroxychloroquine, la dapsone, ou d´autres agents immunomodulateurs [9]. Les cas graves de la maladie, en particulier ceux présentant une glomérulonéphrite ou d´autres formes d'atteinte des organes nobles, peuvent nécessiter des doses élevées de glucocorticoïdes et des agents cytotoxiques. Les agents cytotoxiques de choix sont la cyclophosphamide, la cyclosporine A, l'azathioprine, le mycophénolate mofétil et le methotrexate seul ou en combinaison avec de la prédnisolone. Ces médicaments contrôleront la maladie s´ils sont utilisés à long terme. La colchicine et le rituximab, peuvent être proposés si les lésions sont réfractaires [10]. La plasmaphérèse et la perfusion intraveineuse d'immunoglobuline (IGIV) ont été proposées comme des alternatives valables à considérer en particulier dans les cas d'une détérioration rapide de la fonction rénale ou une glomérulonéphrite rapidement progressive. Dans notre observation, si le traitement par cyclophosphamide, rituximab a permis de contrôler la vascularite sur le plan cutané, il n'a pas eu d'efficacité sur le plan respiratoire (déclin rapide du VEMS). Un traitement par aérosolthérapie à base de salbutamol à la demande et l'association salmeterol/ fluticasone a été instauré avec une nette amélioration clinique.

## Conclusion

L'atteinte pulmonaire au cours de la vascularite urticarienne hypocomplémentémique a plusieurs formes, dont la maladie obstructive qui reste la plus fréquente. Il n'existe à ce jour la aucun traitement spécifique de cette atteinte. La connaissance par les médecins internistes et les pneumologues de cette forme particulière évite de nombreuses complications.
